# REBELOTE, a regulator of floral determinacy in *Arabidopsis thaliana*, interacts with both nucleolar and nucleoplasmic proteins

**DOI:** 10.1002/2211-5463.12504

**Published:** 2018-09-08

**Authors:** Stève de Bossoreille, Patrice Morel, Christophe Trehin, Ioan Negrutiu

**Affiliations:** ^1^ Laboratoire Reproduction et Développement des Plantes Univ Lyon ENS de Lyon UCB Lyon 1 CNRS, INRA Lyon France

**Keywords:** *Arabidopsis thaliana*, ENAP1, NOC, nucleolus, nucleoplasm, OBE1, OBERON, REBELOTE, VFP3

## Abstract

The nucleoplasm and nucleolus are the two main territories of the nucleus. While specific functions are associated with each of these territories (such as mRNA synthesis in the nucleoplasm and ribosomal rRNA synthesis in the nucleolus), some proteins are known to be located in both. Here, we investigated the molecular function of REBELOTE (RBL), an *Arabidopsis thaliana* protein previously characterized as a regulator of floral meristem termination. We show that RBL displays a dual localization, in the nucleolus and nucleoplasm. Moreover, we used direct and global approaches to demonstrate that RBL interacts with nucleic acid‐binding proteins. It binds to the NOC proteins SWA2, AtNOC2 and AtNOC3 in both the nucleolus and nucleoplasm, and also to OBE1 and VFP3/ENAP1. Taking into account the identities of these RBL interactors, we hypothesize that RBL acts both in ribosomal biogenesis and in the regulation of gene expression.

**Abbreviations**
BiFC: bimolecular fluorescent complementation
NOC: nucleolar, complex
RBL: REBELOTE
Y2H: yeast two‐hybrid

The nucleolus is known to be the initial site for ribosomal biogenesis. Processes occurring in the nucleolus include the transcription of ribosomal rDNA by RNA polymerase I and the maturation of ribosomal rRNAs, which are processed and assembled with 5S rRNA and ribosomal proteins to form ribonucleoproteins (RNPs), the precursors of the small and large ribosomal subunits 1. Ribosomal biogenesis involves the regulation of chromatin structure, transcription, RNA processing, and export to the cytoplasm. These processes require a precise spatial organization in the nucleus and numerous protein complexes, including the NOC complexes first isolated in yeast 2, 3.

However, the functions of nucleolar proteins are not restricted to ribosomal biogenesis. Interestingly, several studies have shown the nucleolus to contain proteins unrelated to this process and which are known to be additionally present in other subcellular compartments 4, 5, 6. For example, it has been shown in *Arabidopsis thaliana* that some of these proteins, such as ALY4, are present in the nucleolus and in nuclear speckles, including those corresponding to exon junction complexes, that are involved in mRNA metabolism 6. In addition, it has been shown that some proteins involved in ribosomal biogenesis, including ribosomal proteins, are also involved in specific developmental processes in *A. thaliana* 7, 8. This is the case for SHORT VALVE 1 (RPL24b), which is involved in gynoecium development 9.

Among the proteins involved in ribosomal biogenesis are the NOC2 proteins 3. This family includes nucleolar proteins harboring a broad range of functions in eukaryotes, and as such, it has been the subject of interest for several years. The founding member of this family, *Saccharomyces cerevisiae* ScNoc2p, acts in two protein complexes, together with ScNoc1p and ScNoc3p, two proteins containing a CCAAT‐binding factor (CBF) domain, and acting for the transport of 60S large ribosomal subunits from the nucleolus to the cytoplasm 2. Since this first study, other NOC2 proteins have been found to take part in processes other than ribosomal biogenesis. Among these, the human novel INHAT repressor (NIR) appears to have an (INHAT) function 10. NIR is able to bind nucleosomes and unacetylated core histones and is known to interact with the tumor suppressor p53, and to regulate p53‐target genes by inhibiting histone acetylation. Interestingly, it has been shown that following nucleolar stress, NIR is relocalized from the nucleolus to the nucleoplasm 11. Recently, NIR has also been shown to be necessary for asymmetric cell division in mice, suggesting a requirement in this process for correct mitotic spindle orientation 12. In *Caenorhabditis elegans*, loss of function of the NOC2 homolog causes defects in gonadogenesis 13, whereas in *A. thaliana*, Prunet *et al*. 14 showed that one of the two NOC2 homologs present, REBELOTE (RBL), is involved in floral meristem termination.

In this study, we provide insights on the molecular functions of RBL. We show that this protein is localized in the nucleoplasm and nucleolus, where it acts in NOC complexes similar to those found in yeast that involve the yeast RBL homolog Noc2p, together with the other Noc proteins Noc1p and Noc3p. We also show that NOC proteins from mammals are able to form complexes of similar composition to those in yeast and *A. thaliana*. Moreover, yeast two‐hybrid screens allowed us to show interactions of RBL with nucleic acid‐binding proteins, such as the nucleoplasmic OBE1 and VFP3/ENAP1 proteins, suggesting a role in the regulation of gene expression. In the present work, we have identified the molecular partners of RBL, thus paving the way for further studies to fully elucidate its developmental role.

## Results

### RBL is localized in the nucleolus

**Figure 1:**
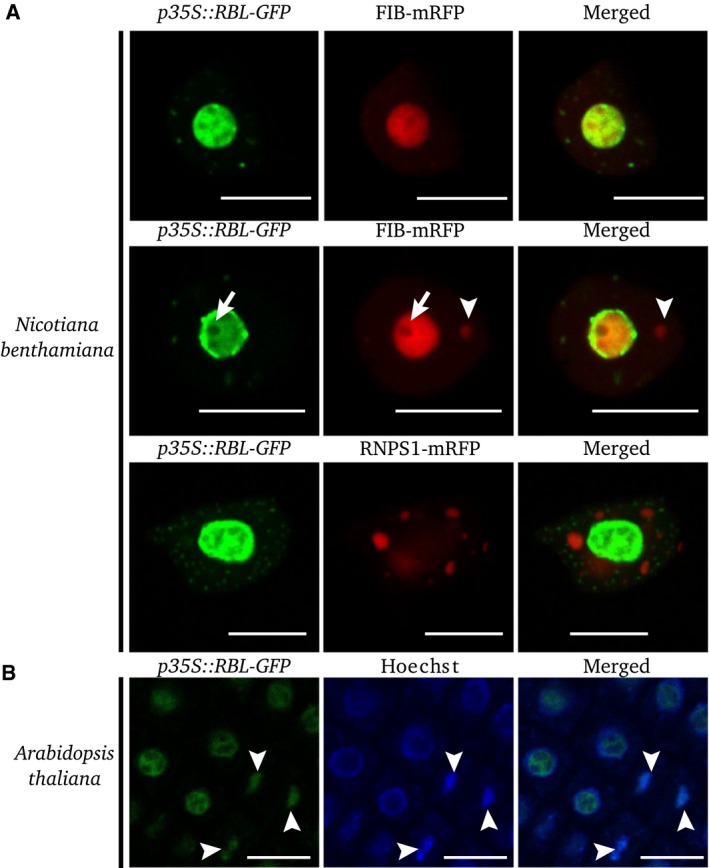
Subcellular localization of REBELOTE. (A) Transient expression of *p35S::RBL‐GFP, with p35S::FIB‐mRFP or p35S::RNPS1‐mRFP* constructs in epidermal cells of tobacco leaves. *p35S::FIB‐mRFP* stains nucleolus and Cajal bodies, whereas *p35S::RNPS1‐mRFP* stains splicing speckles. Arrowheads show the Cajal body stained by FIB‐mRFP chimeric proteins. Arrows show the nucleolar vacuole. (B) Stable transformation of *p35S::RBL‐GFP* construct in *Arabidopsis thaliana*. DNA is stained with Hoechst. RBL colocalizes with DNA during interphase (arrowheads). Scale bar = 10 μm.

Prunet *et al*. 14 showed that RBL was localized in the nucleus. Considering the degree of homology of RBL with yeast Noc2p and human NIR, both of which are localized in the nucleolus despite their involvement in distinct processes in their respective organisms 3, 11, we first addressed the subnuclear localization of RBL. For this purpose, tobacco leaves were infiltrated simultaneously with plasmids encoding the chimeric protein RBL‐GFP, used by Prunet *et al*. 14, and the nucleolus and Cajal body marker FIBRILLARIN (FIB) fused to mRFP. Microscopic observations show that RBL is localized in the nucleolus and nucleoplasm (Fig. 1). However, the nucleolar signal is the stronger and also appears to be heterogeneous. This signal does not completely colocalize with that of FIB‐mRFP and may be more pronounced at the periphery of the nucleolus (Fig. 1A). Both FIB and RBL are absent from the nucleolar vacuole. The nucleoplasmic signal of RBL is much weaker than its signal in the nucleolus and appears to be localized to specific bodies. To identify these RBL‐containing nucleoplasmic bodies, we use FIB‐mRFP and RNPS1‐mRFP, respectively, as markers of Cajal body and splicing speckles 15. Our results show that RBL‐containing bodies are different from Cajal body and splicing speckles (Fig. 1A). The nucleolar localization of RBL was confirmed in *A. thaliana* plants stably transformed with *p35S::RBL‐GFP* or *pRBL::RBL‐mRFP* constructs. As DNA is more abundant in the nucleoplasm than in the nucleolus, we used Hoechst's reagent as a marker of the nuclear compartments. Our data show that RBL is localized in the nucleolus of root cells in interphase, whereas it colocalized with DNA in dividing root cells (Fig. 1B).

### *Arabidopsis thaliana* contains homologs of yeast Noc1p, Noc2p, and Noc3p

Given that RBL has a NOC2 domain and is localized in the nucleolus, we hypothesized that it might interact in nucleolar complexes similar to those in *S. cerevisiae* containing Noc2p together with either Noc1p or Noc3p. Indeed, BLAST searching of the *A. thaliana* genome shows that it contains four *NOC* genes, including a homolog of *Noc1p*, called *SLOW WALKER 2 (SWA2)* 16, two homologs of *Noc2p,* called *AtNOC2* and *RBL,* and a homolog of *Noc3p,* called *AtNOC3*.

**Figure 2:**
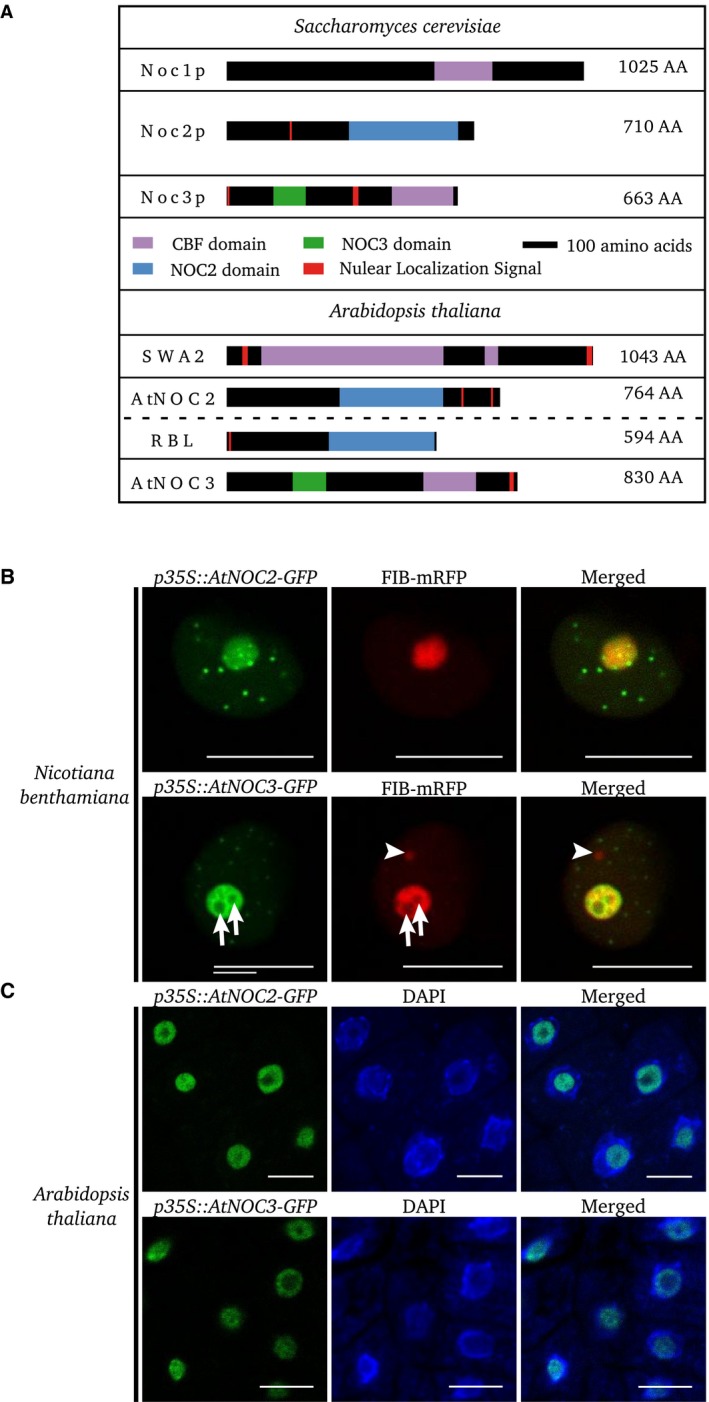
Structure and nucleolar localization of *Arabidopsis thaliana* NOC proteins. (A) Structure of NOC proteins from *Saccharomyces cerevisiae* and *A. thaliana*. (B,C) Transient and stable expression of *p35S::GFP‐AtNOC2* and *p35S::AtNOC3‐GFP* constructs in *Nicotiana benthamiana* (B) and *A. thaliana* (C). Arrowhead shows the Cajal body stained by FIB‐mRFP chimeric proteins, whereas arrows show the nucleolar vacuoles. Observations were performed on tobacco epidermal cells transiently expressing FIB‐mRFP chimeric proteins used as nucleolus and Cajal bodies markers, and on *A. thaliana* root‐tip cells stained with DAPI. Scale bar: 10 μm.

SWA2 is a nucleolar protein of 1.043 amino acids 16, sharing 23% identity and 41% similarity with Noc1p. The protein contains two nuclear localization signals (NLS) at its N and C termini and, as does Noc1p, a CBF DNA‐binding domain (Fig. 2A). RBL and AtNOC2 are 594 aa and 764 aa in length, respectively. As reported by Prunet *et al*. 14, they share 26% identity and 39% similarity. Both proteins encode a NOC2 domain of unknown function. AtNOC2 contains two NLS at its C terminus, whereas RBL has a NLS at its N terminus (Fig. 2A). We also looked for INHAT domains in RBL, AtNOC2, and Noc2p, to determine whether these domains, present in HsNIR, are conserved in other NOC2 proteins. We found homology of the NIR N‐ and C‐terminal INHAT domains, respectively, with the N‐terminal part of AtNOC2 and the C‐terminal region of Noc2p. No INHAT domain was found in RBL. The AtNOC3 protein is composed of 830 amino acids, sharing 21% identity and 36% similarity with Noc3p. Both proteins have a CBF domain, a NOC3 domain of unknown function, and a nuclear localization signal (Fig. 2A).

### AtNOC proteins are coexpressed and display similar subcellular localizations

Next, we attempted to localize AtNOC2 and AtNOC3 at the subcellular level. Observations in infiltrated tobacco leaf cells did not show a complete colocalization of AtNOC2 and AtNOC3 with FIB‐mRFP in the nucleolus (Figs 2B and S1A). Like RBL, AtNOC3 was not present in the nucleolar vacuole (Fig. 2B), and we currently have no evidence for the presence or absence of AtNOC2 in this subnucleolar zone. Both AtNOC proteins are also localized in the nucleoplasm and in nucleoplasmic bodies. The discrete staining patterns of FIB‐mRFP and AtNOC2 and AtNOC3 demonstrate that the nucleoplasmic bodies stained by the latter two proteins cannot be Cajal bodies (Figs 2B and S1A, arrowheads). The nucleolar localizations of AtNOC2 and AtNOC3 were confirmed in transgenic *A. thaliana* root cells, in which we used DAPI to stain DNA (Fig. 2C).

Transcriptomic data from the AtGenExpress database show that the four *A. thaliana NOC* genes are coexpressed at similar developmental stages, while data from ATTED‐II additionally show these genes to be coregulated under similar stress conditions (Fig. S1B) 17, 18. Further to the correlation between *SWA2* and *AtNOC2* expression shown by Li *et al*. 16, we show a correlation between *AtNOC3* and *RBL* expression (Fig. S1C). Moreover, data from ATTED‐II indicate that *AtNOCs* are coexpressed with many genes encoding nucleolar proteins, such as OLIGOCELLULA2, TORMOZ, and APUM23 (Fig. S1D) 19, 20, 21.

### *At*NOC proteins form nucleolar complexes

**Figure 3:**
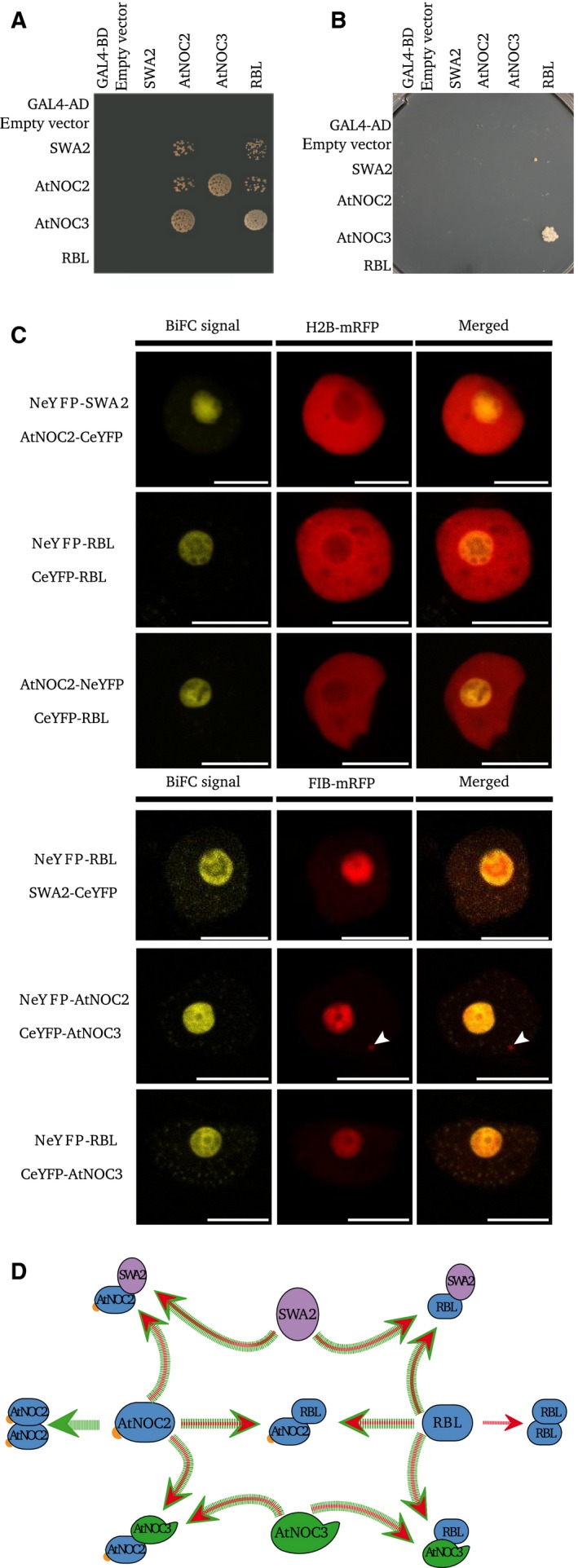
The four *Arabidopsis thaliana* NOC proteins interact in nucleolar complexes. (A) Yeast two‐hybrid interaction matrix on SD‐HTL (10‐3 dilution) selective media. (B) Yeast two‐hybrid interaction matrix on SD‐AHTL (10‐3 dilution) selective media. (C) BiFC experiments using transient coexpression of the nucleoplasmic marker H2B‐mRFP and two *A. thaliana* NOC proteins fused to N‐term or C‐term regions of the YFP fluorescent protein. Arrowhead shows the Cajal body stained by FIB‐mRFP chimeric proteins. Experiments and observations were performed on epidermal cells of *N. benthamiana* leaves. Scale bar: 10 μm. (D) Recapitulative scheme of *A. thaliana* NOC interactions. Yeast two‐hybrid results are in green, BiFC results are in red.

Using yeast two‐hybrid (Y2H) experiments, we evaluated whether the yeast NOC complexes, formed from Noc1p, Noc2p, and Noc3p, are conserved in *A. thaliana*. Interactions were tested two by two, in both directions, by growth on selective media. These assays indicate that SWA2 (the *A. thaliana* Noc1p homolog) is able to bind the two NOC2‐domain‐containing proteins, AtNOC2 and RBL (Fig. 3A). These observations partially confirm the results obtained by Li *et al*. 16, in which SWA2 was shown to interact with AtNOC2, but not with RBL. Similarly, we report interactions of AtNOC3 with both AtNOC2 and RBL. This latter interaction was the strongest observed on SD‐AHTL high selective media (Fig. 3B). Our results are in accordance with the yeast model, in which Noc2p interacts both with Noc1p and Noc3p. Our results demonstrate the heterodimerization of RBL and AtNOC2 and the homodimerization of AtNOC2 (Fig. 3A), which is comparable to the situation in yeast in which the sole homolog of these two A. thaliana proteins, Noc2p, is known to homodimerize 3. Both our results and a published study on the homodimerization of Noc2p in yeast 3 suggest that NOC complexes may be composed of more than two molecules.

With the exception of the homodimerization of AtNOC2 proteins, all interactions found in the Y2H matrix were validated by bimolecular fluorescent complementation (BiFC) (Fig. 3C). Moreover, these BiFC experiments provided evidence of the homodimerization of RBL (Fig. 3C). Cotransformation of RBL‐GFP with H2B‐mRFP or with FIB‐mRFP, respectively, staining the nucleoplasm or the nucleolus and Cajal bodies showed that all interactions took place in the nucleolus and in nucleoplasmic bodies surrounding the nucleolus (Fig. 3C). Our results show that AtNOC2 and AtNOC3 are not interacting in Cajal bodies (Fig. 3C, arrowhead). Moreover, our experiments using FIB‐mRFP as a nucleolar marker tend to confirm that AtNOCs do not have the same subnucleolar localization pattern as FIBRILLARIN (Fig. 3C). Y2H and BiFC results are summarized in Fig. 3D.

### Structural evolution and conservation of NOC complexes in eukaryotes

In addition to the interaction capacities of NOC proteins, we addressed the question of protein function by the use of interspecies complementation tests. *Saccharomyces cerevisiae noc* mutant strains show temperature‐conditional lethality 3. Accordingly, to test their degree of functional redundancy, each *A. thaliana* NOC protein was expressed in its respective *noc* mutant homolog strain. No complementation of lethality was observed at 37 °C (Fig. S2).

**Figure 4:**
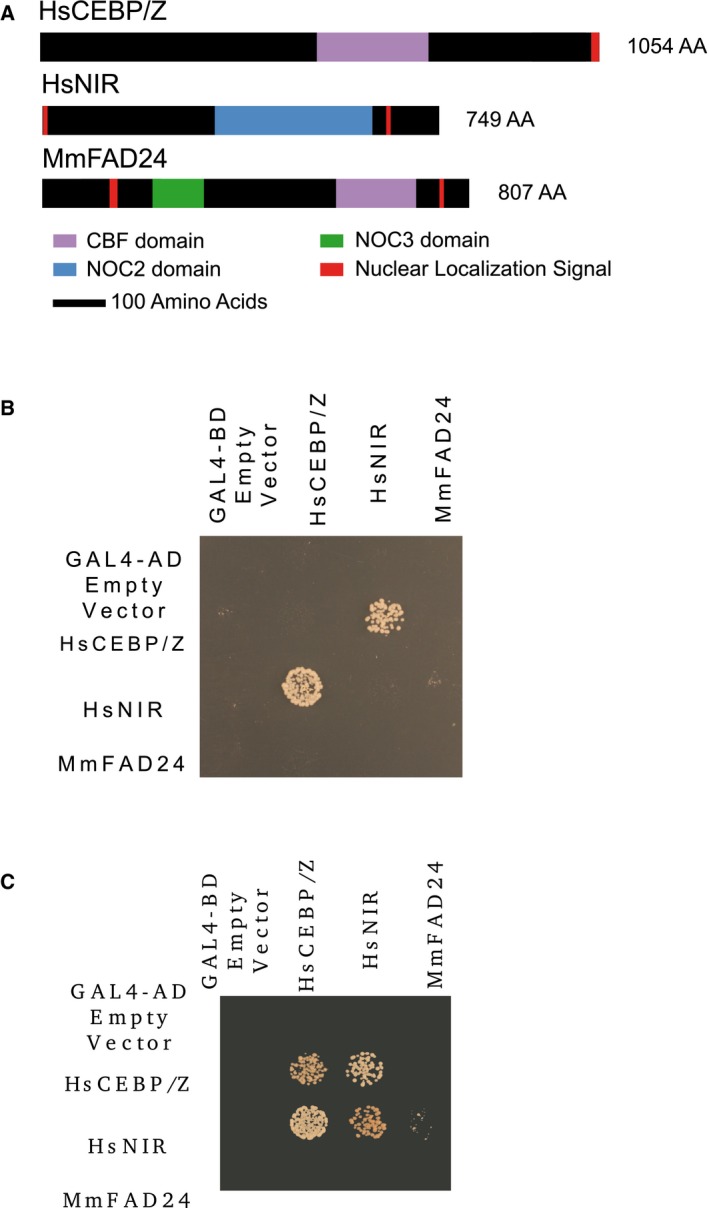
Mammalian NOC proteins and complexes. (A) Schematic representation of HsCEBP/Z, HsNIR, and MmFAD24, which are the respective *Saccharomyces cerevisiae* homologs of Noc1p, Noc2p, and Noc3p. (B) Yeast two‐hybrid interaction matrix on SD‐AHTL (10^−3^ dilution) selective media. (C) Yeast two‐hybrid interaction matrix on SD‐HTL selective media (10^−3^ dilution).

We next addressed the question of the conservation of NOC complexes in mammals, by testing whether NOC proteins from human and mouse are also able to interact *in vitro*. To this end, we used GAL4 fusion proteins containing HsCEBP/Z and HsNIR, the respective Noc1p and Noc2p homologs in humans, and MmFAD24, the Noc3p homolog in mouse, to construct a Y2H interaction matrix. These proteins contain similar domains to their homologs in *S. cerevisiae* and *A. thaliana* (Fig. 4A). We found these mammalian NOC proteins to interact in NOC complexes, as do their yeast and plant homologs. Accordingly, the reciprocal interaction between CEBP/Z and NIR appeared on highly selective SD‐AHTL medium (Fig. 4B). On SD‐HTL selective medium, CEBP/Z and NIR formed homodimers, and NIR interacted with FAD24 (Fig. 4C).

### RBL interacts with OBE1 and VFP3/ENAP1

Given the dual localization of RBL in the nucleolus and the nucleoplasm, we looked for new RBL partners using two independent Y2H screens. Using RBL as bait, more than 1.9 million diploids were plated on high stringency selective medium. Among selected clones, we identified the transcription factors OBE1 (OBERON1) and VFP3/ENAP1 (VirF‐interacting Protein 3/EIN2 nuclear associated protein 1) as putative interactors in both screens. cDNAs encoding VFP3/ENAP1 and OBE1, respectively, represented 40% and 10% of selected diploids.

**Figure 5:**
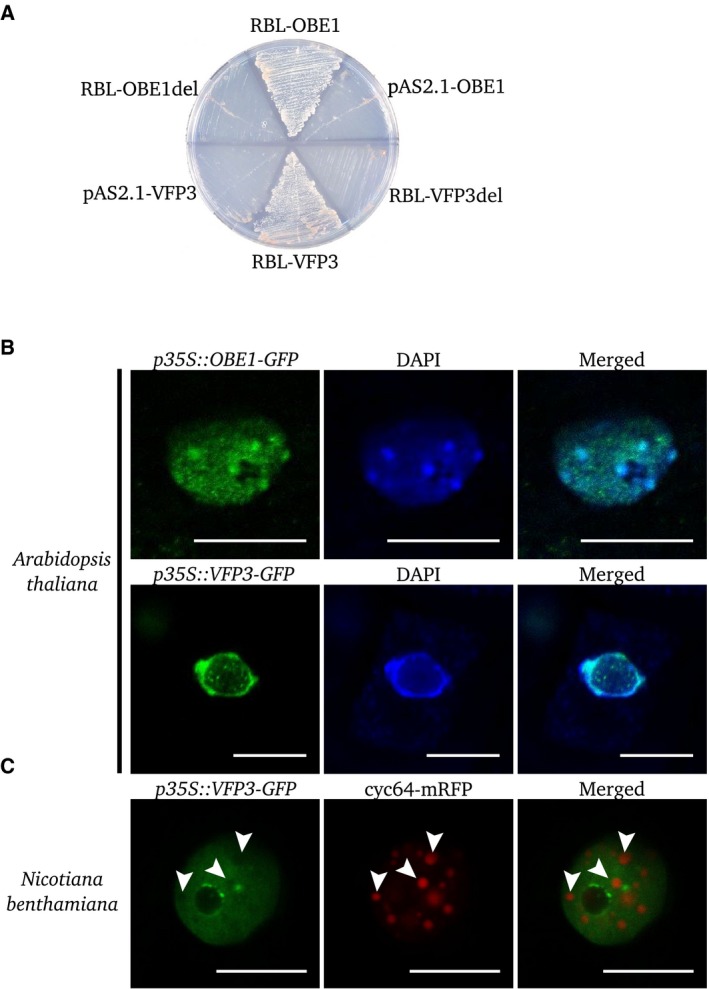
Confirmation of RBL interactors and subcellular localization of interactors. (A) Direct yeast two‐hybrid experiments confirming interactions of RBL with VFP3/ENAP1 and OBE1. Negative controls included tests with empty vectors (pAS2‐1) and with a cDNA in which a nucleotide had been deleted, inducing a frameshift mutation (VFP3/ENAP1del, OBE1del). Yeasts were grown on SD‐AHTL selective media. (B) Localizations of the chimeric proteins OBE1‐GFP and VFP3/ENAP1‐GFP in *Arabidopsis thaliana* root cells. DNA is stained with DAPI. (C) Localization of VFP3/ENAP1‐GFP in tobacco leaf cells. The splicing speckles are stained with cyc64‐mRFP marker. Scale bar: 10 μm.

Yeast plasmid sequencing showed that multiple variable length transcripts of *OBE1* and *VFP3/ENAP1* had been isolated. Confirmation of interactions involving RBL was performed using the Y2H approach. Negative controls using either an empty prey vector or a prey vector containing a cDNA partner inactivated by a frameshift mutation allowed us to confirm the observed interactions of RBL with VFP3/ENAP1 and OBE1 (Fig. 5A).

The OBE1 contains a PHD zinc finger domain (IPR001965) known to act in the regulation of chromatin states 22, 23. OBE1 was characterized as a regulator of apical and root meristem development in the embryo 24, 25. VFP3/ENAP1 belongs to the trihelix family proteins, which includes ASIL1 and ASIL2 that are involved in seed maturation 26, 27. It has been recently shown that VFP3/ENAP1 is involved in ethylene response. VFP3/ENAP1 interacts with EIN2, EIN3, SRT1 and SRT2 and regulates change in histone H3 acetylation levels and ethylene‐dependant transcription 28, 29, 30. Using a GFP transcriptional fusion protein, we investigated the subcellular localization of OBE1 and VFP3/ENAP1. DAPI staining of DNA in transgenic *A. thaliana* root cells showed that OBE1 is a nucleoplasmic protein which is mainly colocalized with heterochromatin, in agreement with the role of OBE1 in chromatin remodeling (Fig. 5B). VFP3/ENAP1 appears distributed in the nucleoplasm and in nucleoplasmic bodies of *A. thaliana* root cells and in transiently transformed tobacco leaf cells (Fig. 5B). Our colocalization experiments with cyc64‐mRFP, a marker of splicing speckles 15, showed that VFP3/ENAP1 nucleoplasmic bodies are not splicing speckles (Fig. 5C, arrowheads). Moreover, VFP3/ENAP1 was occasionally found to be present around the nucleolus (Fig. 5B).

Additionally, to our search for RBL interactors, Y2H experiments were performed to investigate the importance of the NOC2 domain in protein–protein interactions. To that end, the N‐terminal RBL region, containing the NOC2 domain, and its C‐terminal region were used separately as bait in Y2H screens. We found that OBE2, the homolog and partner of OBE1, interacted with the NOC2 domain. No protein was found to interact with the C‐terminal region of RBL.

## Discussion

We report here a functional characterization of RBL, previously reported as being involved in flower meristem determinacy, including its subcellular localization and the identification of some of its interaction partners. Stable transformation of *A. thaliana* and transient expression in *Nicotiana benthamiana* show that RBL is localized in the nucleolus and nucleoplasm and can also be observed in unidentified bodies of nucleoplasm (Fig. 1), a pattern similar to the RBL homologs ScNoc2p from yeast and HsNIR from humans 3, 11.

### RBL interacts in NOC complexes

Interestingly, the NOC complexes, first characterized in yeast, seem to be conserved in diverse organisms including mammals and plants. We show here that the *A. thaliana* genome encodes four NOC proteins that are localized in the nucleolus and nucleoplasm (Figs 1, 2, 3). We present data showing that AtNOCs are not fully colocalized with FIBRILLARIN, a marker of the nucleolar dense fibrillar component (DFC), in which rDNA transcription and the early stages of rRNA processing take place 31. We therefore propose that AtNOCs are localized in a specific compartment of the DFC in which they partially colocalize with FIB and that these proteins are also present in the granular component of the nucleolus, the site enclosing preribosomal particles 31. We furthermore demonstrate that AtNOCs occur in NOC complexes, similar to those observed in *S. cerevisiae,* also containing Noc1p, Noc2p, and Noc3p (Figs 2 and 3) 3. In accordance with results from yeast, our results show that proteins with NOC2‐domain are able to form homodimers and that RBL and AtNOC2 are able to interact together. These data are in partial agreement with published yeast two‐hybrid results that showed that SWA2 interacts with AtNOC2, but not with RBL 16. This difference suggests that the interaction of SWA2 with RBL may be highly sensitive to the expression levels of these proteins in the yeast two‐hybrid experimental system, as Li *et al*. 16 used high‐copy plasmids, whereas we used low‐copy plasmids. Our results also suggest that the NOC2 domain is necessary for interactions involving RBL and AtNOC2. The capacity of NOC proteins to dimerize suggests a molecular and functional diversity of NOC complexes. Such a view is supported by evidence of the coexpression of SWA2 with AtNOC2 and of AtNOC3 with RBL (Fig. S1B). However, AtNOCs have similar nucleolar and nucleoplasmic localization patterns and are coexpressed with other nucleolar proteins, such as OLIGOCELLULA2 and TORMOZ (Fig. S1C), which are homologous to yeast proteins involved in ribosomal biogenesis 19, 21. We therefore hypothesize that *A. thaliana* NOC complexes probably fulfill similar functions to their yeast homologs during the biogenesis of 60S ribosomal subunits 3. A role for RBL in this essential process could explain the embryo‐lethal phenotype observed in *rbl‐3* mutants 14. Furthermore, if RBL is needed for ribosomal subunit biogenesis, the floral meristem determinacy phenotype observed in mutants containing the weak *rbl‐1* allele could be due to a cell homeostasis defect. In addition to the conservation of AtNOC complexes, our results on mammalian NOC interactions (Fig. 4) and complementation tests of yeast mutants by AtNOCs (Fig. S2) suggest the structural coevolution of NOC proteins, which have conserved their ability to interact within their native organisms.

### RBL acts with DNA‐binding proteins

In addition of its nucleolar localization, RBL also displays a nucleoplasmic localization pattern. To gain insight in the role of RBL in the nucleus, we searched for new RBL interactors. Interactions with SWA2, AtNOC3, OBE1, and VFP3/ENAP1, that all contain DNA‐binding domains, suggest that RBL acts to regulate gene expression. A role in gene expression has already been observed for the human homolog of RBL. Through its two INHAT domains, NIR binds to histone tails and inhibits their acetylation by the CBP/p300 acetylation complex, preventing the expression of target genes 10. Our results, showing that AtNOC2 and ScNoc2p share homology with the NIR INHAT domain, suggest that the inhibition of histone acetyltransferase activity by NOC2 proteins might be conserved between major groups of eukaryotes. Despite the absence of an INHAT domain in RBL, we hypothesize that the protein can contribute to the regulation of chromatin structure *via* its protein partners. Recent publications show that VFP3/ENAP1 acts in histone H3 acetylation 28, 29, 30, and we might therefore hypothesize that, *via* its binding with VFP3/ENAP1, RBL interferes with histone H3 acetylation. Indeed, RBL localization at nucleoplasmic dots could be linked to the regulation of post‐translational histones modifications. Similarly, RBL could act on chromatin remodeling *via* its partners OBE1 and OBE2 proteins, which contain PHD zinc fingers domain reported to act in this regulatory process 22. The chromatin‐related function of the OBE1/OBE2 complex is supported by the localization in present work of OBE1 at DNA‐condensed loci and by our Y2H experiments showing the interaction of OBE1 with MOM1 (Fig. S3), a plant‐specific major regulator of heterochromatin loci 32. The interaction of RBL with OBE1 and OBE2 could provide clues on the floral meristem phenotype observed in *rbl* 14. OBE1 and OBE2 have indeed been shown to regulate embryo meristem initiation and apical meristem maintenance 24, 25, 33. From these and previous studies, we can hypothesize that the OBE1/OBE2 complex also promotes floral meristem maintenance and that RBL binds to the OBE1/OBE2 complex to inhibit this activity, thereby leading to floral meristem termination. As OBE1 and OBE2 are redundant, it has been necessary to study the functions of these genes in double‐mutant context 24, 25. However, the embryo lethality of the *obe1 obe2* double mutant did not allow us to study the role of OBE1/OBE2 in floral termination. To shed light on the role of the RBL/OBE1/OBE2 complex in floral termination process, further studies should be focused on inducible RNAi or CRISPR technology to target both OBE paralogs *in planta*.

Although RBL, OBE1, and VFP3/ENAP1 are shown in the current work to possess distinct localization patterns. The capacity of RBL to interact with these two other proteins may be explained by a possible dynamic protein relocalization between the nucleolus and the nucleoplasm. This phenomenon has been clearly documented for numerous other nuclear proteins 11, 15, 34. Furthermore, NIR, the human homolog of RBL, is known to relocalize to the nucleoplasm after nucleolar stress 11. It should also be mentioned that the homolog of VFP3/ENAP1 in tobacco, NtSIP1, which is also localized in the nucleoplasm, has been shown to interact with the nucleolar protein NtSIP2 35. Such a relocalization may be a response to pathogens and/or environmental stimuli. Similarly, we know that RBL's partners OBE1 and OBE2 act in responses to virus infection through interactions with potyvirus VPg protein 36 and that VFP3/ENAP1 acts in ethylene responses, in addition to its interaction with the VirF oncoprotein of *Agrobacterium tumefaciens,* responsible for crown gall tumors 28, 29, 30, 37, 38.

## Conclusion

Our study demonstrates that RBL is both a member of NOC complexes and a cofactor interacting with DNA‐binding protein. We suggest that RBL is involved, by participating in different protein complexes, in multiple cellular processes, from ribosomal biogenesis to the regulation of gene expression, and thus joins a growing list of nucleolar proteins that control specific developmental processes, such as floral meristem termination 7, 8. Our work also raises the question of the role of nucleolar proteins on floral meristem termination. Interestingly, the *A. thaliana* nucleolar protein NUCLEOSTEMIN‐like 1, which is involved in ribosomal biogenesis, also controls floral meristem termination 39, 40. Its yeast homolog, Nug1p, interacts with Noc2p to export 60S ribosomal subunit from the nucleus 41, 42, 43. Accordingly, our work lays the foundations of future studies on the functions of NOC2 proteins and on the relationship between the nucleolus and developmental processes in plants.

## Materials and methods

### Localization and bimolecular fluorescent complementation

We used the previously described construction *p35S::RBL‐GFP* for RBL localization 14. Other cDNAs used for localization experiments were cloned in‐frame with a GFP reporter gene in Gateway pK7FWG2 and pK7WGF2 vectors (Ghent Gateway Vectors). Localization observations were made either on transiently transformed *N. benthamiana* leaves or on roots of *A. thaliana,* stably transformed by the floral dip method. For BiFC, full‐length cDNAs of *RBL* and its interactors were cloned in the pBiFP1‐4 Gateway vectors developed by F. Parcy (CEA, Grenoble, France). Infiltrations were performed on 3‐week‐old *N. benthamiana* leaves as previously described 44. Observations were made immediately after infiltration.

### Complementation of yeast *noc* mutants

Yeast *noc1‐1*,* noc2‐1,* and *noc3‐1* mutants and vectors expressing *ScNoc* were provided by H. Tchoschner 3. *Arabidopsis thaliana* cDNAs were cloned in the YEplac195 Gateway expression vector, also provided by H. Tchoschner. Thermo‐sensitivity complementation was tested using nondiluted and diluted yeast colonies cultured at 37 °C on SD‐U medium.

### Yeast two‐hybrid experiments

A cDNA library of etiolated *A. thaliana* seedlings (Stock CD4‐22: https://www.arabidopsis.org/servlets/TairObject?type=stock%26id=88570) was initially screened using full‐length *RBL* cloned in pAS2.1 (Clontech). The cDNA library was cloned in the pADGAL4‐2.1 vector 45. Y2H experiments were performed by mating, using *S. cerevisiae* AH109 and Y187 strains and selection on triple (‐Leu, ‐Trp, ‐His) or quadruple (‐Ade) selective media 41. Computer‐based sequence analysis to identify putative interactors was performed using VectorNTI (Informax) and sequencher (Gene Codes Corporation, Ann Arbor, MI, USA) software.

The interaction of RBL with OBE1 and VFP3 were confirmed by mating between AH109 (carrying pADGAL4‐2.1 and full‐length cDNAs of interactors, or cDNAs containing frameshift mutations caused by deletions) and Y187 (carrying the empty pAS2.1, or pAS2.1 and RBL cDNA) 46.

For direct Y2H experiments using *A. thaliana* proteins, full‐length cDNA were cloned into the pPC86‐ and pPC97‐based vectors. *NIR was* amplified from a human cDNA library obtained from the LBMC laboratory (ENS, Lyon). *CEBPZ was* amplified from the *pMT2‐CBF* construct 47, while the *FAD24* cDNA was amplified from the *pGFP‐c1‐mouseFAD24* plasmid 48, provided by Pr. Masayoshi Imagawa (Nagoya City University). For Y2H experiments using mammalian proteins, cDNAs have previously been cloned in the pENTRY vector pDONRzeo and then transferred by recombination in pGBKT7‐GW and pACT2‐GW destination vectors (developed by Annie Chaboud, ENS, Lyon).

### Microscopy

Imaging in subcellular localizations and in BiFC experiments was performed using Zeiss LSM‐510, Zeiss LSM‐700, or Leica SP5 confocal microscopes. A histone H2B‐mRFP marker was kindly provided by Frederic Berger 49, while FIB‐mRFP, cyc64‐mRFP, and RNPS1‐mRFP were kindly provided by P. Shaw 15.

### Bio‐informatic prediction

We used online tools to study coexpression of AtNOC proteins and predict the presence of nuclear localization signal or specific protein domain. We respectively used ATTED‐II 17 and AtGenExpressed 18 for expression analysis, NLS‐mapper 50 for NLS prediction, and INTERPRO 51 for protein domain prediction.

## Accession numbers

RBL (At3g55510), SWA2 (At1g72440), AtNOC2 (At2g18220), AtNOC3 (At1g79150), OBE1 (At3g07780), VFP3/ENAP1 (At3g11100)

## Author contributions

SdB, PM, CT, and IN conceived and designed the project. SdB and PM acquired the data. SdB, PM, CT, and IN analyzed and interpreted the data. SdB, PM, CT, and IN wrote the manuscript.

## Supporting information


**Fig. S1.** AtNOC2 localization and co‐expression of *A. thaliana* NOC. (A) Localization of AtNOC2‐GFP and FIB‐mRFP in tobacco leaf cell. Arrowheads show the Cajal bodie. Scale bar: 10 μm. (B–D) Graphical representation of *AtNOC* co‐expression from micro‐array data using the ATTEDII and AtGenExpress tools. (B) Specific co‐expression using abiotic stressbased micro‐arrays. (C) Global co‐expression using developmental stage‐based micro‐arrays. (D) Co‐expression network of *AtNOC* genes.
**Fig. S2.** Absence of complementation of yeast noc mutants by *A. thaliana* NOCs. Full‐length *AtNOC, ScNOC* and *CRABS CLAW (CRC)* were expressed in yeast mutants *noc1‐1* (A), *noc2‐1* (B) and *noc3‐1* (C). Complementation of the lethal phenotype at 37°C is observed by yeast growth with or without dilution. *CRC* was used as negative control and yeast Noc proteins as positives controls for their respective mutants.
**Fig. S3**. OBE1 interacts with MOM1 in yeast. Yeast two‐hybrid interaction matrix on SD‐AHTL selective media (10‐3 dilution). This is a confirmation of results of a yeast two‐hybrid screen using OBE1 as bait, in which we isolate MOM1 cDNA.
