# Supplementary material for: REBELOTE, a regulator of floral determinacy in Arabidopsis thaliana, interacts with both nucleolar and nucleoplasmic proteins
Source: FEBS Open Bio. 2018 Sep 8;8(10):1636–48. doi: 10.1002/2211-5463.12504 (PMC6168688; doi:10.1002/2211-5463.12504)
Supplement: Supplementary file 1 — Fig. S1. AtNOC2 localization and co‐expression of A. thaliana NOC. (A) Localization of AtNOC2‐GFP and FIB‐mRFP in tobacco leaf cell. Arrowheads show the Cajal bodie. Scale bar: 10 μm. (B–D) Graphical representation of AtNOC co‐expression from micro‐array data using the ATTEDII and AtGenExpress tools. (B) Specific co‐expression using abiotic stressbased micro‐arrays. (C) Global co‐expression using developmental stage‐based micro‐arrays. (D) Co‐expression network of AtNOC genes. Fig. S2. Absence of complementation of yeast noc mutants by A. thaliana NOCs. Full‐length AtNOC, ScNOC and CRABS CLAW (CRC) were expressed in yeast mutants noc1‐1 (A), noc2‐1 (B) and noc3‐1 (C). Complementation of the lethal phenotype at 37°C is observed by yeast growth with or without dilution. CRC was used as negative control and yeast Noc proteins as positives controls for their respective mutants. Fig. S3. OBE1 interacts with MOM1 in yeast. Yeast two‐hybrid interaction matrix on SD‐AHTL selective media (10‐3 dilution). This is a confirmation of results of a yeast two‐hybrid screen using OBE1 as bait, in which we isolate MOM1 cDNA. [file FEB4-8-1636-s001.pdf]

Fig. S1 AtNOC2 localization and co-expression of A. thaliana NOC

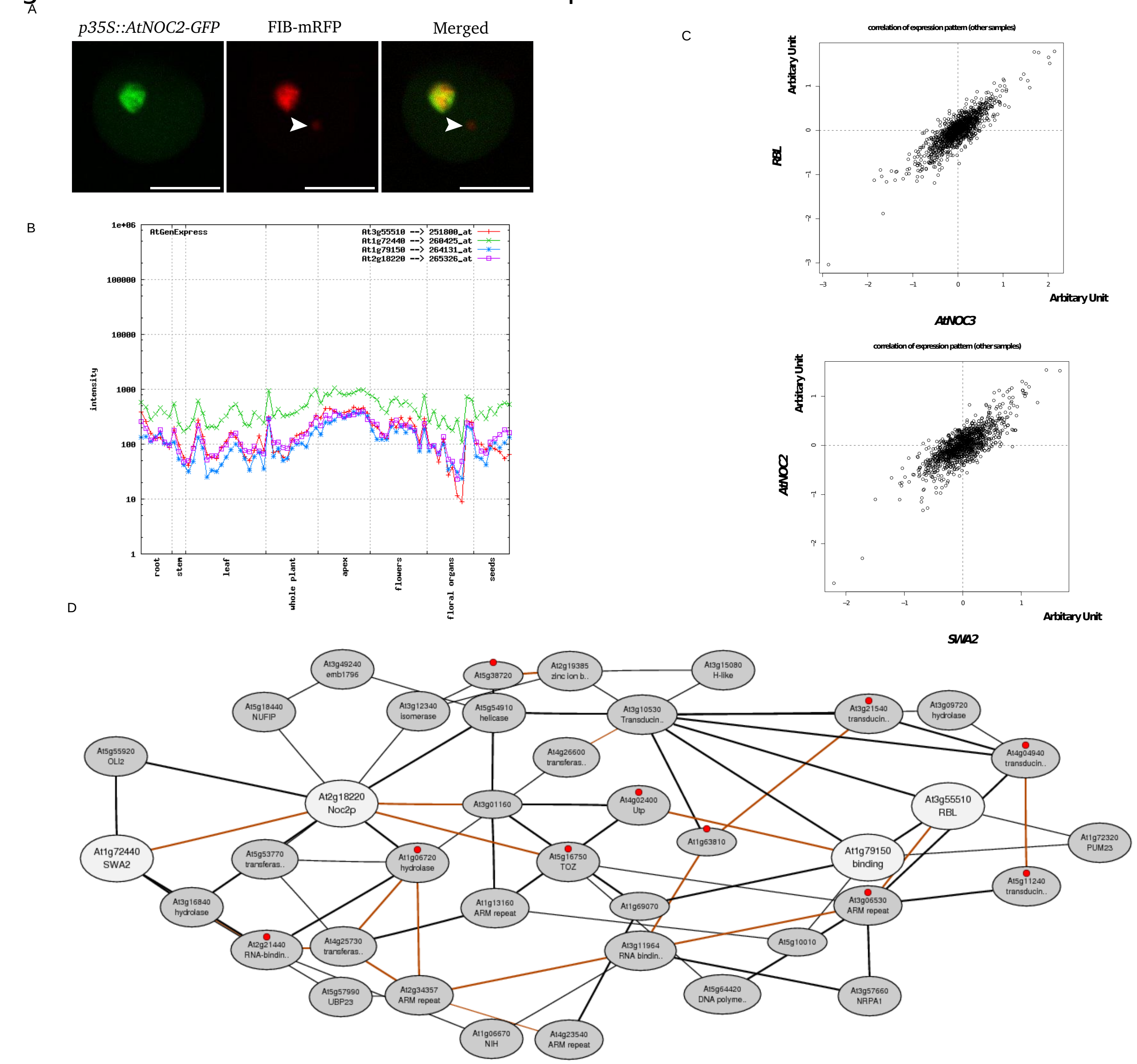

Fig. S2 Absence of complementation of yeast noc mutants by A. thaliana NOCs

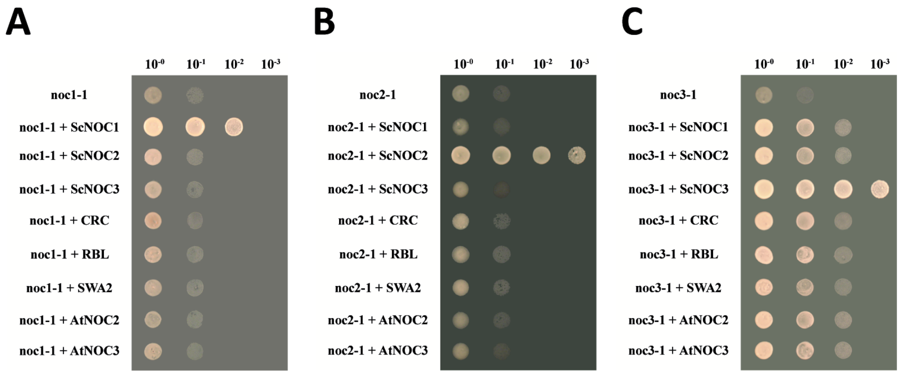

Fig. S3 OBE1 interacts with MOM1 in yeast

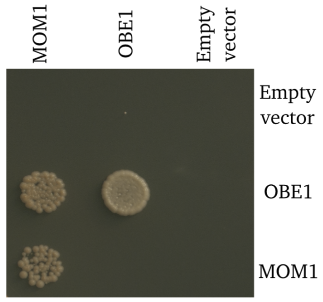

Supporting information captions

Fig. S1: AtNOC2 localization and co-expression of *A. thaliana NOC*.

(A) Localization of AtNOC2-GFP and FIB-mRFP in tobacco leaf cell. Arrowheads show the Cajal bodie. Scale bar: 10 µm. (B-D) Graphical representation of *AtNOC* co-expression from micro-array data using the ATTEDII and AtGenExpress tools. (B) Specific co-expression using abiotic stress-based micro-arrays. (C) Global co-expression using developmental stage-based micro-arrays. (D) Co-expression network of *AtNOC* genes.

Fig. S2: Absence of complementation of yeast *noc* mutants by *A. thaliana* NOCs.

Full-length *AtNOC*, *ScNOC* and *CRABS CLAW (CRC)* were expressed in yeast mutants *noc1-1* (A), *noc2-1* (B) and *noc3-1* (C). Complementation of the lethal phenotype at 37°C is observed by yeast growth with or without dilution. *CRC* was used as negative control and yeast Noc proteins as positives controls for their respective mutants.

Fig. S3: OBE1 interacts with MOM1 in yeast

Yeast 2-hybrid interaction matrix on SD-AHTL selective media (10-3 dilution). This is a confirmation of results of a yeast 2-hybrid screen using OBE1 as bait, in which we isolate MOM1 cDNA.
